# Quantifying mangrove carbon assimilation rates using UAV imagery

**DOI:** 10.1038/s41598-024-55090-w

**Published:** 2024-02-26

**Authors:** Javier Blanco-Sacristán, Kasper Johansen, Mariana Elías-Lara, Yu-Hsuan Tu, Carlos M. Duarte, Matthew F. McCabe

**Affiliations:** 1 Climate and Livability Initiative, Division of Biological and Environmental Sciences and Engineering, King Abdullah University of Science and Technology, 23955-6900 Thuwal, Saudi Arabia; 2 Red Sea Research Center and Computational Bioscience Research Center, King Abdullah University of Science and Technology, Thuwal, Saudi Arabia

**Keywords:** Image processing, Climate-change ecology, Ecophysiology, Ecosystem services, Forestry, Restoration ecology

## Abstract

Mangrove forests are recognized as one of the most effective ecosystems for storing carbon. In drylands, mangroves operate at the extremes of environmental gradients and, in many instances, offer one of the few opportunities for vegetation-based sequestering of carbon. Developing accurate and reproducible methods to map carbon assimilation in mangroves not only serves to inform efforts related to natural capital accounting, but can help to motivate their protection and preservation. Remote sensing offers a means to retrieve numerous vegetation traits, many of which can be related to plant biophysical or biochemical responses. The leaf area index (LAI) is routinely employed as a biophysical indicator of health and condition. Here, we apply a linear regression model to UAV-derived multispectral data to retrieve LAI across three mangrove sites located along the coastline of the Red Sea, with estimates producing an R^2^ of 0.72 when compared against ground-sampled LiCOR LAI-2200C LAI data. To explore the potential of monitoring carbon assimilation within these mangrove stands, the UAV-derived LAI estimates were combined with field-measured net photosynthesis rates from a LiCOR 6400/XT, providing a first estimate of carbon assimilation in dryland mangrove systems of approximately 3000 ton C km^−2^ yr^−1^. Overall, these results advance our understanding of carbon assimilation in dryland mangroves and provide a mechanism to quantify the carbon mitigation potential of mangrove reforestation efforts.

## Introduction

Mangrove forests occupy tidal mudflats, river banks and coastlines across tropical and subtropical marine environments around the world. Even though mangroves are a high-priority ecosystem for conservation and offer a wide range of ecosystem services worldwide^1^, they have also suffered from degradation and deforestation^2^. Over the period from 1980 to 2000, it was estimated that approximately 35% of the world’s mangrove forests disappeared^3,4^, leaving only 15.2 million hectares remaining^5^. Indeed, the loss rate of mangroves was higher than the average loss of vegetation seen in other tropical and subtropical forests, with losses being detected in 97% of all countries and territories surveyed. Considering the rapid decline in mangrove extent over the last 50 years^6^, a trend that is slowing down in many regions but increasing in others^7^, mangrove forests must be protected to avoid their functional disappearance in the next 100 years^7,8^.

Apart from their primary role as ecosystem engineers, mangroves are recognized as being among the most efficient of all ecosystems at fixing and storing carbon^9,10^. Together with seagrasses and saltmarshes, the amount of carbon that mangrove forests remove is significantly higher than other ecosystems such as temperate and tropical forests on an area equivalent basis^11^. Mangroves occupy just 0.5% of the global coastal area, yet contribute 10–15% of coastal sediment carbon storage, while exporting 10–11% of the particulate terrestrial carbon back to the ocean^12^. Although the carbon stock of mangroves can differ significantly in relation to the tidal range and topography of their local environment^13^, at the global scale, the average whole-ecosystem carbon stock in mangroves has been estimated at around 95,600 ton C km^−2^^12^. However, this value includes the carbon stored both above- and below-ground, and includes that contained within the soils of mangrove forests. As such, it is not representative of the carbon assimilated by photosynthesis, which is variable and largely dependent on environmental factors, and which is the focus of this investigation.

Although most of the carbon stored in mangroves appears in the soil and below-ground pools of dead roots^6^, mangrove photosynthesis converts atmospheric carbon into organic compounds that are ultimately used to develop storage reserves and new plant tissue, as well as to develop chemical defences^12^. While maximum carbon assimilation rates in mangrove leaves can exceed 25 μmol C m^−2^ s^−2^, average values range from 5 to 20 μmol C m^−2^ s^−1^^14^. Because mangroves appear in anoxic, salty soils, they have developed mechanisms to maximize carbon assimilation by developing physiological plasticity, thus improving transpiration and water use efficiency depending on the surrounding environmental conditions^15,16^. However, rates of carbon assimilation, defined here as net photosynthesis and measured in units of μmol C m^−2^ s^−1^, vary widely among species, with vapor pressure deficit, light availability and intensity, and soil salinity, all playing a regulatory role^17^. These environmental factors overlay the typically nutrient-limited nature of the intertidal areas that mangroves occupy^18^ and in combination, can cause mangroves to develop defense mechanisms that act to reduce their carbon assimilation. For example, photo-inhibition (i.e., light-dependent loss in photosynthesis) takes place when more light is absorbed than can be used in photosynthetic photochemistry^19^. Under these conditions, excessive light can be dissipated as heat, which can damage the photosynthetic chemistry of the leaves, resulting in changes in carbon assimilation. Furthermore, assimilation rates are maximal at leaf temperatures ranging from 25 to 35 °C, with significant declines in efficiency observed beyond leaf temperatures of 35 °C (e.g.,^15^). During periods of intense insolation, even at optimal leaf temperatures, the transpiration rates are not sufficient to prevent heating of the leaves above ambient air temperatures, causing a decline in carbon assimilation^20^. These environmental stressors usually reduce the mangrove carbon assimilated in arid regions, where temperatures can be extreme and precipitation low^21^, compared to mangroves in more humid parts of the world that are richer in nutrients^12^.

Seasonal rates of carbon assimilation vary greatly and, depending on the mangrove species, exhibit different behaviour. Although mangroves are less prone to phenological changes compared to other plant types, there is significant seasonal variation in annual carbon fluxes, with higher values in winter, when the environmental conditions are conducive to vegetation health. Within this seasonality, rainfall variability can be more influential in the physiology of mangrove trees than low precipitation itself^22^. In arid regions, which exhibit some of the highest interannual precipitation seasonality in the world, rainfall variability is further increased^23^. Among arid regions, hyperarid environments, such as the Red Sea, present little seasonality, with extremely high temperatures and low, if not absent, precipitation throughout much of the year. Low values of humidity are known to reduce photosynthetic carbon gain^13^, but the humidity in the Red Sea region is continuously high. Hence, while the environmental conditions within the Red Sea may appear unfavorable for the growth of mangroves, the consistent absence of seasonal fluctuations in both rainfall and temperature, coupled with a sustained high humidity level, could potentially establish a dependable setting for mangroves to sequester carbon. This environment would be devoid of the challenges posed by irregular rainfall patterns and humidity shits encountered in other arid regions characterized by greater seasonality where mangroves appear.

Developing tools and techniques that allow for the determination of carbon assimilated by mangroves throughout the year in a timely and cost-effective manner is needed. Eddy-covariance methods^24^ are most commonly used to measure carbon assimilation in mangroves^25–27^, but these methods require the deployment of expensive infrastructure, depend on wind conditions and cover relatively small areas. Light attenuation methods to measure carbon assimilation tend to relate the amount of light absorbed by the mangrove canopy to the total canopy chlorophyll content^28,29^. Methods following this principle often use measurements of the leaf area index (LAI) together with average rates of carbon assimilation to calculate the net daytime canopy photosynthesis^29,30^. LAI is well recognized as one of the most important biophysical parameters for assessing vegetation health^31^ and has been used as a key descriptor of biological and physical processes such as respiration and nutrient cycling^32,33^. As LAI constitutes a key trait for quantifying and monitoring carbon exchange^30,34^, an accurate assessment of LAI provides indirect insight on the state of mangroves. As such, changes in LAI can be used as an indicator for changes in vegetation carbon exchange^35^. For example,^36^ used LAI measurements to estimate the carbon assimilated by mangrove forests in Pichavaram, India, while^37^ evaluated mangrove carbon exchange from LAI measurements collected along the arid coast of Western Australia. However, previous research has estimated carbon assimilation from LAI using field-based measurements, which are typically difficult and time-consuming to obtain.

The retrieval of physical properties of mangrove forests can be challenging to characterize due to the intricate and challenging architecture of mangroves, their complex root networks, and their diurnal tidal inundation. To alleviate these issues, an increasing number of studies have utilized unmanned aerial vehicles (UAVs) to estimate structural properties that can be related to carbon assimilation in mangroves, such as biomass using LiDAR data (e.g.,^38–40^) or photogrammetric methods including structure from motion techniques (e.g.,^41^). Leaf pigments, such as chlorophyll a and b, and their concentration, have also been a focus of UAV-based applications^42–44^. Although several studies have estimated LAI using both UAV-based multispectral (e.g.,^45,46^) and hyperspectral imagery (e.g.,^47,48^), none has yet studied the feasibility of using LAI imagery to estimate net photosynthesis in mangroves.

At the canopy level, carbon assimilation by photosynthesis is affected by physical parameters of the stands, such as the canopy area and the LAI^49^. Carbon assimilated through photosynthesis is often used as a proxy for the CO_2_ assimilated by an ecosystem, particularly by vegetation^50^. In this context, we hypothesize that UAV-derived LAI can be used to accurately quantify carbon assimilation rates of mangrove ecosystems. To test this hypothesis, the objectives of this work were: (a) to use field and UAV-based multi-spectral data to estimate the LAI of mangroves, and (b) employ the UAV-derived LAI together with leaf-level measurements of gas exchange to map the carbon assimilation of mangroves.

## Data and methodology

### Study sites

**Figure 1:**
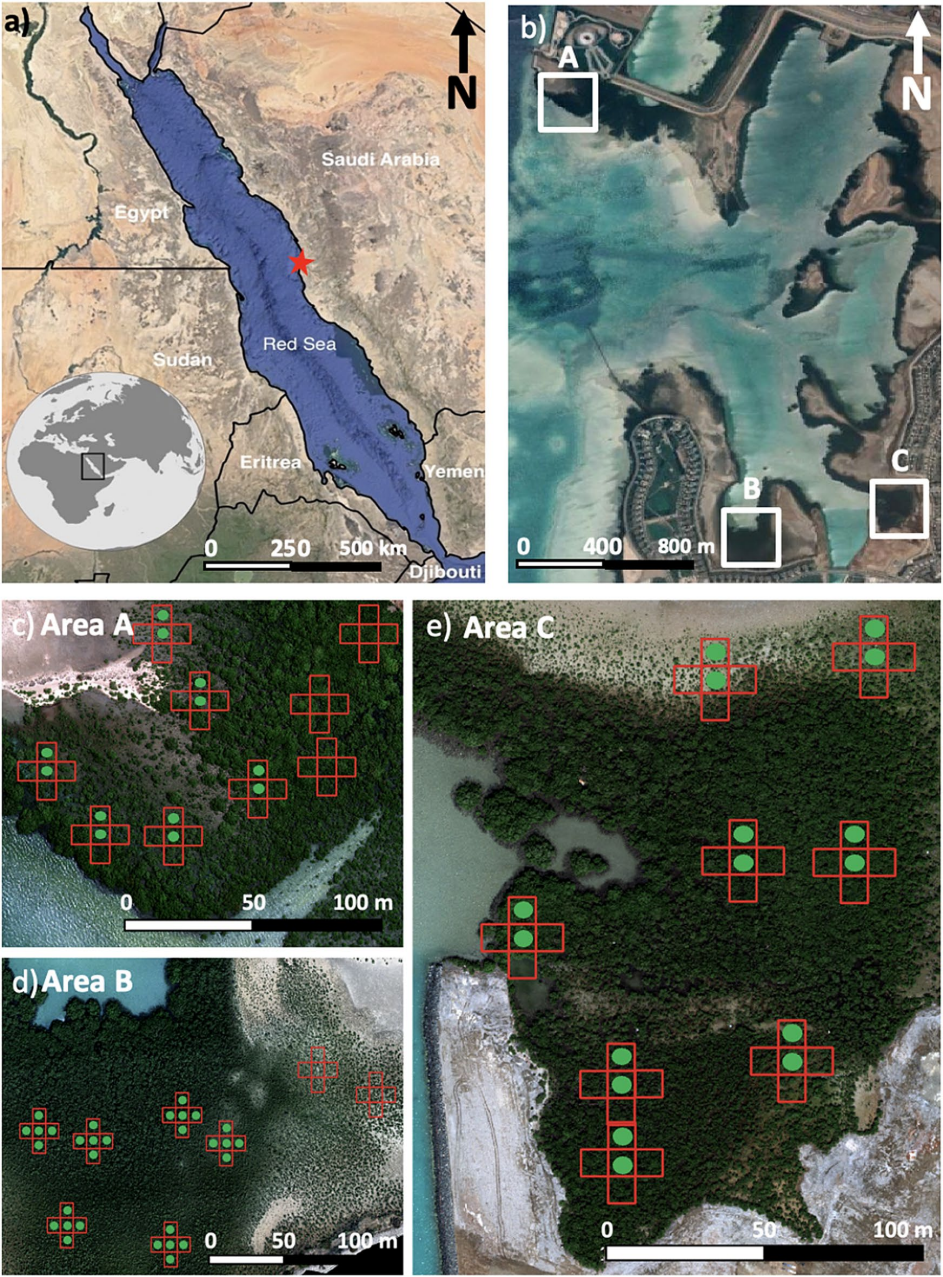
(**a**) Approximate location of the study sites along the central Red Sea coast; and (**b**) details of a satellite view of the three field locations. The locations of sampling plots within the three field sites are overlain on high spatial resolution UAV images (**c**–**e**), designated as Area A, B and C, respectively. A 5-unit measurement grid was employed at each site, with nine sampling points identified for Area A, and eight for Areas B and C. The individual red squares from this grid represent 10 × 10 m sub-plots where coincident LAI measurements were collected ("Leaf area index measurements" section). The green dots represent the location of field-collected net photosynthesis measurements ("Measuring net photosynthesis" section).

The central Saudi Arabian coastline of the Red Sea is characterized by a tropical hot arid climate, with an annual rainfall of less than 100 mm^51^. The study focuses on several mangrove sites located on the Red Sea coastline, one of the most environmentally extreme areas in the world supporting mangrove growth. Three mangrove study sites, consisting of the dominant *Avicennia marina* species in the region and located in geographical proximity to one another were identified (Fig. 1)*.* Due to concerted local-scale afforestation efforts, the extent and density of the mangrove stands have increased over the last decade, and each of the sites exhibits a range of mangrove densities, with tree heights varying from the sapling stage to approximately 3 m tall.

### Data collection

Three separate data collection campaigns were undertaken across three different seasons, i.e. in summer (June 30, 2021) for Area A, in autumn (October 28, 2021) for Area B and in winter (February 15, 2022) for Area C, combining coincident collections of UAV-based multi-spectral imagery ("Unmanned aerial vehicle data" section) with ground-based LAI ("Leaf area index measurements" section) and leaf-level net photosynthesis measurements ("Measuring net photosynthesis" section). During each field campaign, the ground-based data were collected within 10 × 10 m sub-plots. The locations of these plots were selected based on a UAV-derived NDVI map to ensure that the full range of canopy densities was covered. The three selected study areas consisted of only *Avicennia marina* and were located within 2 km of each other. The major differences observed between the mangrove stands in each area were their tree density and height, largely attributable to differing tree ages. Hence, we assumed that data collected from one area at a specific time would be indicative of physiological conditions in the other areas. More detailed information regarding the specific elements of the data collection and analysis framework is provided in the sections below.

#### Unmanned aerial vehicle data

Multispectral images of the study sites were collected using a MicaSense RedEdge-MX camera system (MicaSense Inc., Seattle, WA, USA). The MicaSense camera, which captures data in five spectral bands (475, 560, 668, 717, 842 nm) was mounted on a DJI Matrice 100 quadcopter (SZ DJI Technology Co., Ltd, China). All flights were performed during clear sky conditions and with low wind speeds, with flight planning undertaken using the Universal Ground Control Station (UgCS) software (SPH Engineering, Latvia; Vers. 4.6). The UAV was flown at 100 m ASL at a speed of 6 m/s, with a side distance of 18 m between flight lines and forward and side overlaps of 91% and 79%, respectively. The camera collected images at nadir every 1 s, with camera exposure for each band set manually to ensure brightness consistency and preclude saturation of the photos. Ground control points (GCPs) were distributed throughout the study site in order to geo-reference the UAV orthomosaics. Areas A, B and C had 5, 9 and 15 GCPs deployed, respectively. In Area 1, only 5 GCPs were deployed due to the high density of the mangroves, making identification of the GCPs from above difficult. GCP coordinates were measured via a Leica GS10 base station with an AS10 antenna and a Leica GD15 smart antenna as a rover (Leica Geosystems, St. Gallen, Switzerland). Radiometric calibration of the collected imagery was assisted via six near-Lambertian panels in white, four shades of grey, and black^52^, which were placed in the field and measured with an ASD FieldSpec-4 spectrometer (Malvern Panalytical, Malvern, UK). The center of each plot was marked in the field using a bright aluminum disc with a diameter of 50 cm for subsequent visual identification in the UAV imagery.

The UAV imagery was processed using the Agisoft Metashape Pro software (Agisoft LLC, St. Petersburg, Russia) to produce a georeferenced multispectral orthomosaic and a digital surface model (DSM) with a pixel size of 7 cm. A digital terrain model (DTM) was generated by filtering out non-ground points from the dense point cloud. A canopy height model (CHM) was derived by subtracting the DTM from the DSM. A vicarious radiometric correction (also called sensor-information-based calibration;^53–56^) was applied to create orthomosaics of surface reflectance. While the linear empirical correction is the most widely used radiometric correction approach to convert UAV data to at-surface reflectance, the vicarious correction has proven to provide more accurate results while also reducing the number of at-surface reflectance pixels occurring with negative values^57^. The vicarious radiometric correction considers photograph parameters, such as the exposure time, to compensate the brightness variation of the images and convert their digital numbers directly to spectral radiance or surface reflectance, depending on whether simultaneous irradiance measurements are available. The equation for the vicarious radiometric correction used herein, as provided by AgEagle Sensor Systems Inc. (2021), is described as:

$$L = V(x,y) \times \frac{{a_{1} }}{g} \times \frac{\rho - \rho BL}{{t_{e} + a_{2} y - a_{3} t_{e} y}}$$

where *L* is the spectral radiance in W/m^2^/sr/nm; *V*_*(x,y)*_ is the vignetting polynomial function; *g* is the sensor’s gain, *t*_*e*_ is the image exposure time; *ρ* is the normalized raw pixel values; *ρ*_*B*L_ is the normalized black current value; and *a*_1–3_ are the calibration coefficients. Once the spectral radiance is calculated, surface reflectance can be derived by dividing the radiance with the simultaneous irradiance measurements. The signal-to-energy conversion needs at least one known reflectance panel for normalization. In this case, a panel with a surface reflectance of around 20% was used^43^. This reflectance correction method was implemented when generating the orthomosaic.

#### Leaf area index measurements

Leaf area index (LAI) measurements were acquired using a LiCOR LAI-2200C instrument (LI-COR Biosciences, Nebraska, USA). The instrument measures LAI based on the attenuation of the diffusive sky radiation at 490 nm. Two optical sensor wands were operated simultaneously to collect mangrove LAI measurements during dawn and dusk. A black round cap with a 90° gap was placed on both sensors to (i) only measure light from that specific 90° gap of the near-hemispheric view; (ii) avoid scattering radiation, and (iii) block out the direction of the operator holding the wand. The sensor measuring above-canopy radiation (Sensor A) was fixed and leveled on a tripod in an open area within 100 m of the mangroves. In this case, the 90° gap was directed towards the north to avoid any incoming radiation from the Sun and measurements were recorded once every 30 s. With another sensor (Sensor B), five below-canopy readings for each 10 × 10 m plot were collected, with a measurement taken in the center and at 2.5 m from the center in north, south, east and west directions. During the measurements, Sensor B was positioned as low as possible to the ground to maximize the distance from the sensor to the leaves and oriented towards the north to ensure consistency with the above canopy measurements. Data from both Sensors A and B were post-processed using the FV2200 software (LI-COR Biosciences, Nebraska, USA) to calculate the LAI and relevant statistics from each plot. The five LAI measurements collected within each 10 × 10 m plot were averaged, resulting in a total of 119 LAI values (39, 40 and 40 values collected in Areas A, B and C, respectively).

#### Measuring net photosynthesis

The LI-6400/XT instrument (LI-COR Biosciences, Nebraska, USA) was used to measure net photosynthesis within the mangroves. The LI-6400/XT instrument included a leaf chamber fluorometer (6400–40 LCF) (which was set as the only light source) and a CO_2_ mixer. The LI-6400/XT has been used previously to measure leaf-to-air gas exchange in mangroves and is considered the gold standard for such measurements (e.g.,^58–60^). By calculating the difference in the composition of gas in the chamber before and after light exposure to the leaf, the instrument can measure the carbon exchange between the leaf and the air^61^. Using an infrared gas analyzer, H_2_O and CO_2_ concentrations of incoming air are measured prior to the air being cooled or warmed by the system. Once measured, the air is delivered to the leaf cuvette (which hosts the leaf of interest) and a mixing fan is used to circulate the air throughout the cuvette and a second infrared gas analyzer cell. By measuring the differences in concentrations of H_2_O and CO_2_ in the air before and after the exposure of the leaf, the variables of interest (i.e., net photosynthesis in our case) can be calculated. Readers are directed to^61^ for a more detailed description of the LI-6400XT system. Based on the instrument configuration, a measurement of the net photosynthesis rate, measured in μmol CO_2_ m^−2^ s^−1^, was derived for each leaf sample. The LI-6400/XT instrument was deployed at 12 sub-plots in Area A, 30 sub-plots in Area B, and 16 sub-plots in Area C (Fig. 1). Within the 10 × 10 m sub-plots, five leaves from trees deemed representative (based on size and leaf density) were selected and measured using the following protocol: (1) health, determined by visual inspection; (2) young but fully expanded, determined by coloration; and (3) sun-facing at the time of the measurements to ensure the leaves were already active. The measurements were collected throughout the day from early in the morning until sunset and selected to ensure a representative range of forest densities within each area.

### Methodology

#### Determining LAI through multiple linear regression

Using the five multispectral bands available from the MicaSense camera, several vegetation indices were calculated based on the derived orthomosaics (see Table 1) in order to relate them to the field-measured LAI from the study areas. To ensure representative values of these indices on a per-plot basis, the pixel level values were averaged over the 10 × 10 m sub-plots (see Fig. 1). A dataset containing the in-field LAI values and the corresponding averaged values of the UAV-derived vegetation indices from each sub-plot was created.

**Table 1:** UAV-derived variables calculated from the multispectral orthomosaics.

Variable	Abbreviation	Calculation	References
Blue normalized difference vegetation index	blueNDVI	$\frac{{\rho }_{842}-{\rho }_{475}}{{\rho }_{842}+{\rho }_{475}}$	^62^
Green normalized difference vegetation index	greenNDVI	$\frac{{\rho }_{842}-{\rho }_{560}}{{\rho }_{842}+{\rho }_{560}}$	^62^
Normalized difference red edge index	NDRE	$\frac{{\rho }_{842}-{\rho }_{717}}{{\rho }_{842}+{\rho }_{717}}$	^63^
Normalized difference vegetation index	NDVI	$\frac{{\rho }_{842}-{\rho }_{668}}{{\rho }_{842}+{\rho }_{668}}$	^64^
Near-infrared red edge and red index	NIR.RE.Red	$\frac{{\rho }_{842}+{\rho }_{717}{-\rho }_{668}}{{\rho }_{842}+{\rho }_{717}+{\rho }_{668}}$	^65^
Red edge and green ratio	RE.Green	$\frac{{\rho }_{717}-{\rho }_{560}}{{\rho }_{717}+{\rho }_{560}}$	
Red edge normalized difference vegetation index	RENDVI	$\frac{{\rho }_{717}-{\rho }_{668}}{{\rho }_{717}+{\rho }_{668}}$	^65^
Canopy height model	CHM	DSM—DTM	

A multiple linear regression model, i.e. the *lm* function implemented in R (R Core Team, 2020), was created using the UAV data (i.e., spectral bands, VIs and the CHM) and field-derived LAI measurements from all three areas. To assess the performance of this model, the coefficient of determination (R^2^) and root mean square error (RMSE) between the predicted LAI and field-collected LAI measurements were calculated. In addition, the model was assessed by calculating the cross-validated statistics following the leave-one-out cross-validation procedure. The importance of each variable to the model was calculated as the predictor variables’ relative contribution in the multiple linear regression model using the *calc.relimp* function from R’s *relaimpo* package^66^. This regression model combining the measurements from all three study areas was used to produce spatial maps of LAI for each individual study area. These LAI maps were necessary for the consequent spatial carbon assimilation assessment.

#### Estimating carbon assimilation in mangroves

Daily carbon assimilation in the mangroves was derived by combining the field-measured net photosynthesis and the UAV-derived LAI maps to calculate net canopy photosynthesis (P_N_). We decided to use LAI for carbon assimilation quantification because LAI, as a direct measure of the leaf area per unit ground area, is strongly related to the photosynthesis of vegetation, and hence serves as a good proxy for quantifying carbon exchange^31^. While other indicators such as vegetation cover, biomass, or tree height provide valuable structural information, LAI integrates aspects of vegetation structure directly related to its photosynthetic function and, consequently, carbon exchange capacity^35^. In addition, LAI can be highly sensitive to environmental conditions, such as light, temperature, and water availability, which makes it a useful indicator for assessing the impact that environmental changes or stressors might have on carbon exchange. Following^29^, P_N_ (μmol CO_2_ m^−2^ s^−1^) was calculated as *P*_*N*_ = *A* ∗ *d* ∗ *LAI***,** where *A* represents the average net photosynthesis (μmol CO_2_ m^−2^ s^−1^) from the collected measurements ("Measuring net photosynthesis" section), *d* represents the daylight hours (calculated here as the average day length of the year in the study area; i.e. 11.6 h) and *LAI* represents the leaf area index (m^2^/m^2^) of each pixel from the UAV-derived LAI maps (see "Determining LAI through multiple linear regression" section). As mentioned in^29^, this method provides a measure of the amount of carbon assimilated by net photosynthesis in the canopy during daylight hours and has been shown to yield better results than other methods (e.g.,^29,36^). Based on the resolution of the UAV orthomosaics, the native pixel size of 51.41 cm^2^ was used to convert P_N_ from μmol CO_2_ m^−2^ s^−1^ to kg C pixel^−1^ yr^−1^ to ultimately obtain the unit weight of carbon assimilated per pixel in the UAV images (Equation S1). The total carbon assimilated per year for each area was calculated using the total number of pixels in the UAV orthomosaics belonging to mangroves, identified as pixels with empirically derived thresholds of NDVI > 0.1, NIR > 0.1 and Red < 0.2.

## Results

### Deriving LAI from UAV data

**Figure 2:**
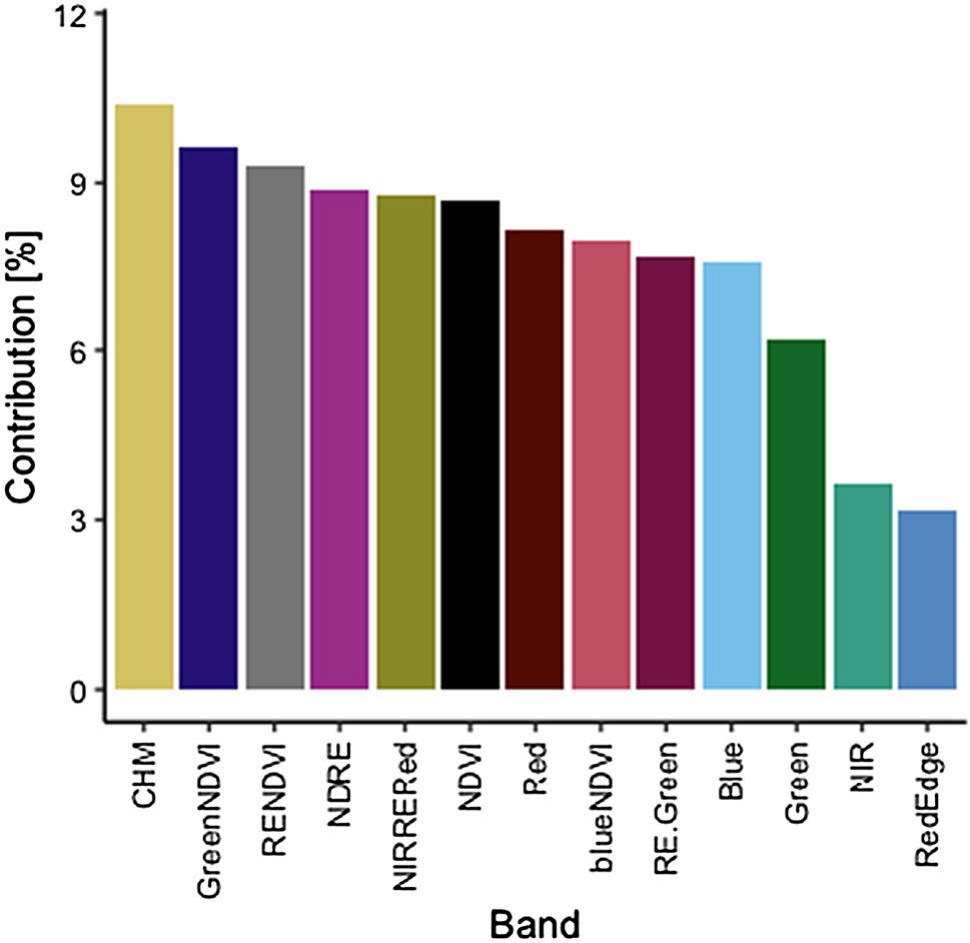
Variable importance, calculated as the predictor variables relative contribution in the multiple linear regression model, based on the unmanned aerial vehicle (UAV) image dataset for the prediction of leaf area index (LAI).

A multiple linear regression model was used to estimate LAI by relating the UAV-derived metrics (Table 1), consisting of five spectral bands, seven vegetation indices and the canopy height model, with the ground-measured LAI measurements collected from the 119 sub-plots across the three study areas. The 119 measurements of field-measured LAI exhibited minimum and maximum values of 0.19 and 4.34, respectively. Seven out of thirteen variables had importance values of between 8 and 10.4%, indicating that no variable had a really high contribution to the model.s can be seen in Fig. 2, the CHM was the variable with the highest importance (10.40%), while the NIR and RedEdge bands had the lowest importance (3.61% and 3.18%, respectively). The height heterogeneity among the mangrove stands within the three study areas might explain the higher importance of the CHM for predicting LAI among the three sites. Conversely, it is known that NIR and RedEdge bands are highly sensitive to chlorophyll concentration and not to structural parameters in mangroves, such as the LAI (e.g.,^46,62^), which may explain their low importance values in the model.

**Figure 3:**
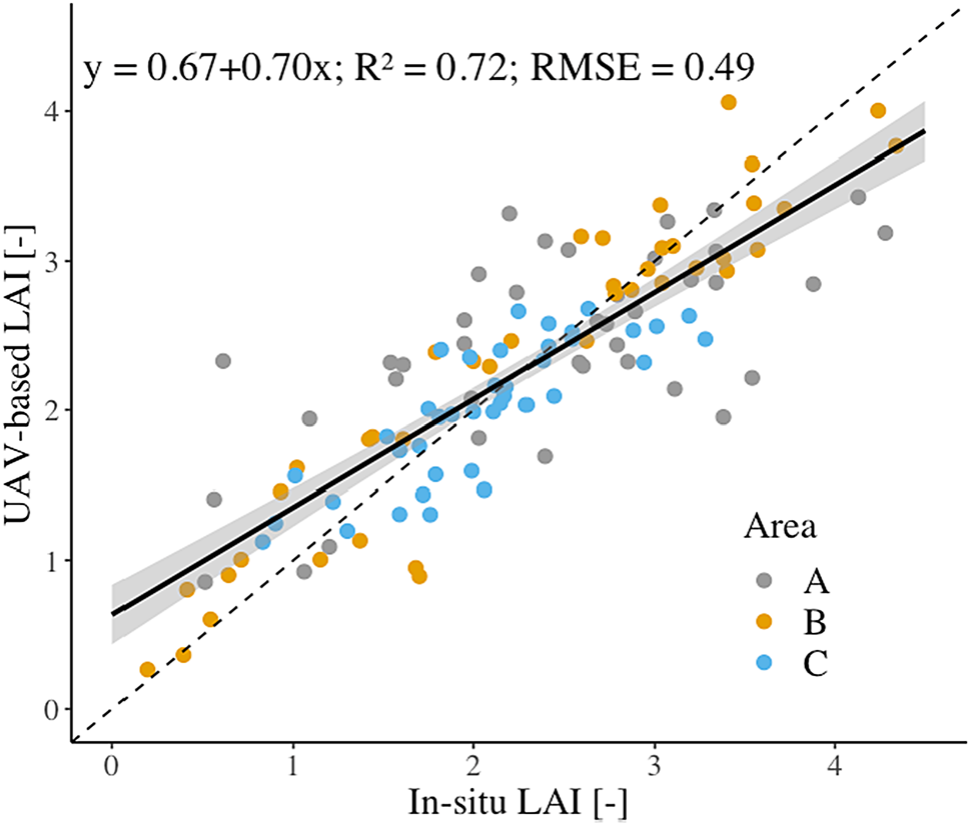
Scatterplot showing the relationship between field-measured and predicted LAI based on unmanned aerial vehicle (UAV)-based multispectral data from the three study areas. The shaded area represents a ± 95% symmetrical confidence interval, calculated from the standard errors of the predicted values. The dashed line represents the 1:1 line.

The relationship between the field-measured LAI and the UAV-predicted LAI produced an R^2^ value of 0.79 (Fig. 3), indicating that a single model can be developed for predicting LAI across the different mangrove locations. The statistics in cross-validation presented very similar values to the original analysis, with an R^2^ of 0.71 and a RMSE of 0.50. Although most of the points are located around the 1:1 line, the relationship shows both over- and under-estimation of in-field measured LAI. These deviations can be related to some of the sub-plots from Area A with low tree heights (i.e. CHM values) but dense stands (i.e. high LAI values), which affected the relationship between in-field and UAV-derived LAI.

### Mapping the spatial distribution of carbon assimilation

**Figure 4:**
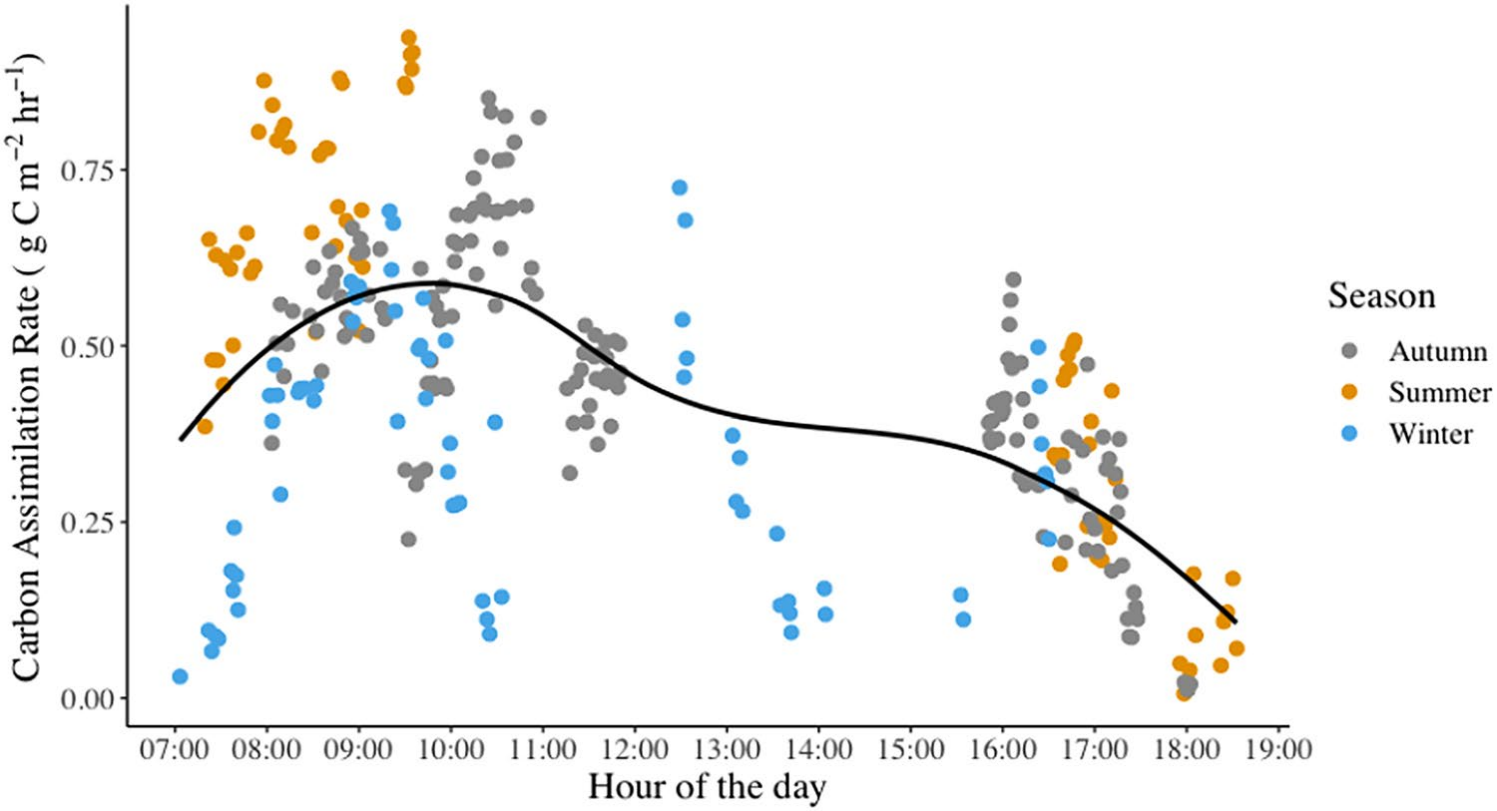
Carbon assimilation rates measured at leaf level in the mangroves of the three study zones at different times of the year: summer (Area A), autumn (Area B) and winter (Area C). The black line represents the fitted curve of the data. Note that summer and autumn datasets lack records between 12:00 and 16:00 because of very high ambient air temperatures, which precluded field sampling between those times.

After developing the relationship between in-field and UAV-based LAI, we employed the UAV-derived LAI maps (Fig. S1) to calculate the carbon assimilation of the mangrove stands. The field measurements of net photosynthesis showed daily variability (Fig. 4), with the highest values in the first morning hours and the lowest values at the end of the day. However, at the beginning of the day, the highest photosynthesis rates were measured in Area A (summer) and the lowest in Area C (winter), which likely reflect the highest and lowest temperatures throughout the year, respectively. These net photosynthesis measurements provided a mean value of 0.45 g C m^−2^ h^−1^ (i.e., 10.35 μmol CO_2_ m^−2^ s^−1^) within the three study areas, in line with estimates published in previous studies of *A. marina* (e.g.,^36,67^). Using this mean net photosynthesis value, we can then exploit the spatially distributed maps to expand beyond the point-sampled locations, providing insights into other local mangrove areas occurring in similar environmental settings.

**Figure 5:**
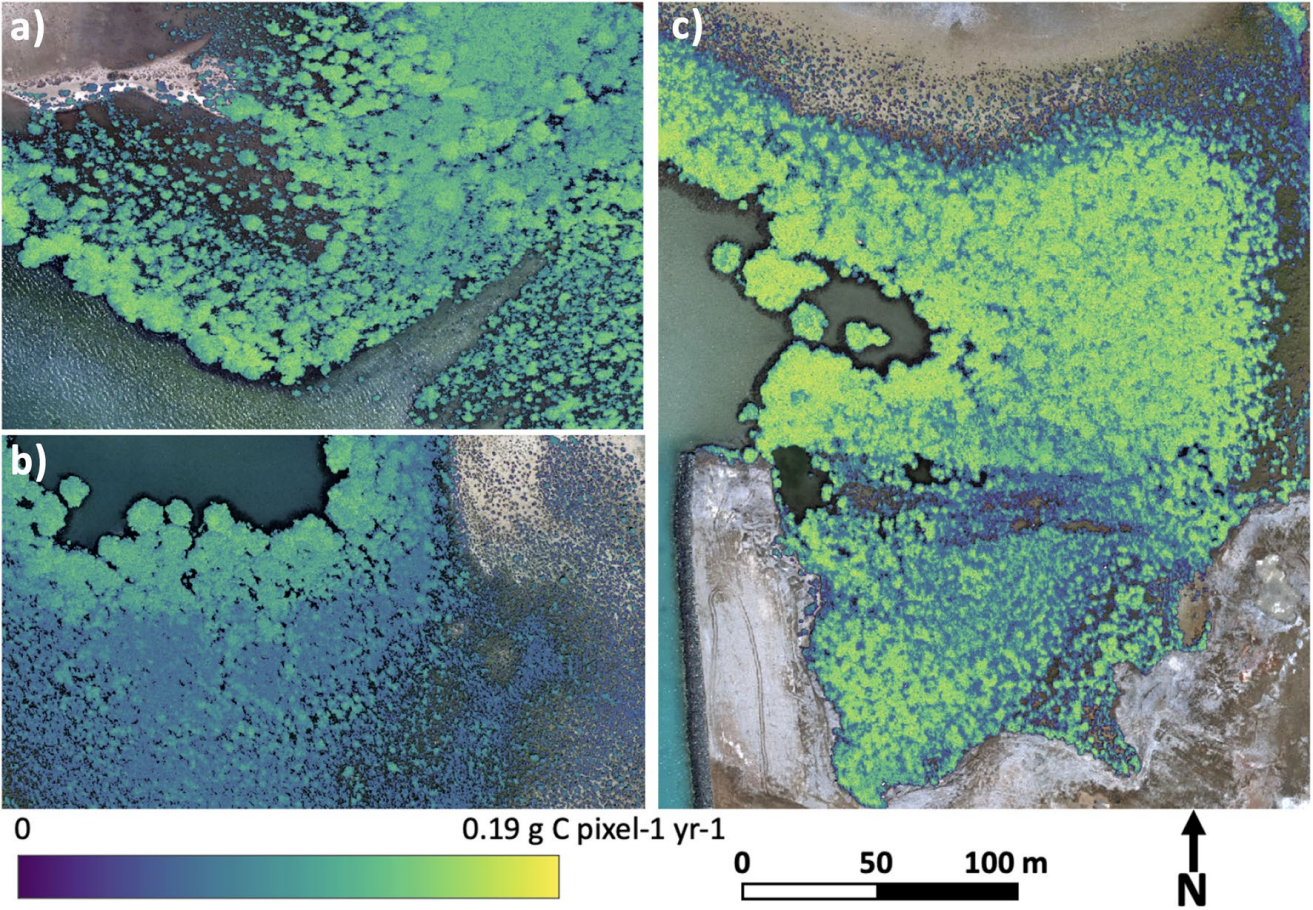
Maps of estimated carbon assimilation per pixel and year obtained from leaf area index measurements predicted from UAV imagery of a subset of: (**a**) Area A; (**b**) Area B; (**c**) Area C.

Maps of the spatial distribution of carbon assimilation in the three study areas were generated from the average net canopy photosynthesis, the average daylight duration and the UAV-derived LAI (Fig. 5). As can be seen, the map for Area C has greater spatial variability, reflecting a coefficient of variation of 76.91% derived from the in-field net photosynthesis measurements. Conversely, Area A shows a more homogeneous distribution of carbon capture, with a coefficient of variation of 38.68% determined from its in-field net photosynthesis measurements. Based on these carbon assimilation maps, we can determine the total carbon assimilated in each mangrove area per year, with values of 400.8 ton C yr^−1^ for Area A (0.24 km^2^), 224. On C yr^−1^ for Area B (0.09 km^2^) and 194.1 ton C yr^−1^ for Area C (0.04 km^2^). These values correspond to carbon assimilation rates of 1670.1 ton C km^−2^ yr^−1^, 2488.9 ton C km^−2^ yr^−1^, and 4851.3 ton C km^−2^ yr^−1^ for Areas A, B and C, respectively, representing an average carbon assimilation of 3003.4 ton C km^−2^ yr^−1^. The different carbon assimilation values among the studied stands, which are within a few kilometers of each other, might be explained by the different densities of each stand. These different densities are expressed by the varying LAI values, which are ultimately used to produce the final carbon assimilation maps ("Estimating carbon assimilation in mangroves" section).

## Discussion

Our upscaled results for carbon assimilation rates of 3003.4 ton C km^−2^ year^−1^ based on LAI maps, are consistent with previous studies from other geographical locations derived using eddy-covariance methods, albeit from mangrove species different to *A. marina* (Table 2). Using eddy-covariance methods,^26^ reported mangrove carbon assimilation rates of 1271 ton C km^−2^ yr^−1^ in Sunderbans, India, while^68^ estimated 2305 ton C km^−2^ yr^−1^ in Pichavaram, India. Also using eddy-covariance methods,^25,69^ reported carbon assimilation rates in Florida between 2190 and 2759 ton C km^−2^ yr^−1^.^27^ reported values of 1451 and 1668 ton C km^−2^ yr^−1^ in two different semiarid mangrove stands in the Gulf of California. Studies using eddy-covariance systems benefit from continuous measurements, even at midday, when conditions for taking measurements using gas-exchange chambers are challenging in our study area, particularly in summer, when temperatures often exceed 40° at midday. Our species of study, *A. marina*, shows varying carbon assimilation rates, as observed in New Caledonia, where^70^ found that dwarf (tree heights lower than 0.6 m) *A. marina* stands assimilate carbon at a rate of 974 ton C km^−2^ yr^−1^. In Hong Kong,^72^ reported assimilation rates of 2784 ton C km^−2^ yr^−1^, similar to that observed in our work.

**Table 2:** Mean values of carbon assimilation rates (CAR; T C km^−2^ yr^−1^) and photosynthesis rates (A; g C m^−2^ h^−1^) from mangroves in different geographical regions.

Location	Species	Climate	Height (m)	Method	CAR	A	References
Sunderbans, India	Mixed stands of *Aviccenia alba*, *Bruguiera* *gymnorrhiza* and *Rhizophora* spp.	Tropical moist	5–6	EC	1271	NA	^26^
Pichavaram, India	Mixed stands of *Rhizophora* spp. And *Avicennia* spp.	Subhumid	3–7.5	EC	2305	NA	^68^
Florida, USA	Mixed stands of *Rhizophora mangle*, *Laguncularia racemosa* and *Aviccenia germinans*	Tropical monsoon	15–20	EC	2190	NA	^69^
Florida, USA	*Rhizophora mangle*, *Laguncularia racemosa* and *Aviccenia germinans*	Tropical monsoon	NA	EC	2759	NA	^25^
Gulf of California, USA	Mixed stands of *Rhizophora mangle*, *Laguncularia racemosa* and *Aviccenia germinans*	Dry arid	4	EC	1559.5	NA	^27^
Coeur de Voh, New Caledonia	*A. marina*	Semi-arid	0.6	EC	974	NA	^70^
Hong Kong	*A. marina*	Subtropical monsoonal	6.5	EC	2784	NA	^71^
Kala Oya, Sri Lanka	Mixed stands of *B. gymnorrhiza, L. racemose, R. mucronata, A. marina, B. cylindrica, E. agallocha, C. tagal, A. corniculatum*	Tropical wet	7.11	Point-scale gas exchange chamber		0.43	^67^
Tamil Nadu, India	*A. marina*	Tropical wet	4.34	Point-scale gas exchange chamber		0.47	^36^
Red Sea, Saudi Arabia	*A. marina*	Tropical hot arid	2	Point-scale gas exchange chamber	3003.4	0.45	This study

*EC* eddy-covariance, *NA* not available, *A* photosynthesis rate.

Several factors need to be considered in evaluating these results. Rainfall variability, which is known to negatively affect mangrove physiology^72^ and hence, their carbon assimilation, is minimal to non-existent in our study area. As the humidity and temperature in our study area (central Red Sea coast of Saudi Arabia) remain high throughout the year, it is not a stress factor, as observed in mangroves located in other arid regions of the world^13^. The lack of seasonality regarding humidity, rainfall and the persistently high temperatures introduces a degree of stability in the environmental conditions throughout the year, which favors mangrove development. Further, the photosynthesis measurements reported here were collected from healthy and sun-facing leaves from well-established, tall mangroves (> 3 m). Mangrove tree height and age have been found to be closely and positively related to the amount of assimilated carbon^26,36^. Therefore, due to the selection of healthy leaves and the absence of large fluctuations in environmental factors, our derived estimates of carbon assimilation might represent the upper range, rather than the average, of assimilation values for *A. marina* stands within our study areas.

Rates of carbon assimilation in mangroves can become saturated at high sunlight irradiation levels due to the photosystem response to excessive solar input^73–75^. In addition, carbon assimilation capacity tends to decrease with high salinity levels^76,77^, which can reduce stomatal conductance and/or carboxylation. However, evidence suggests that defense mechanisms in *A. marina,* such as reducing chlorophyll levels and increasing leaf angles, are exacerbated by environmental controls, such as high light intensities and salinities^74,77,78^. The adaptability of *A. marina* to extreme conditions reflects the net photosynthesis results described in this work (i.e., 0.45 g C m^−2^ h^−1^), which are similar to those from other *A. marina* stands in more humid regions, including Sri Lanka (67; 0.43 g C m^−2^ h^−1^; annual mean humidity of ~ 80%) and India (36; 0.47 g C m^−2^ h^−1^; annual mean humidity of ~ 73%). Such an outcome lends some support to the idea that *A. marina* may be better adapted to dry conditions than *Rhizophora mucronata*, the other mangrove species present in the Red Sea region^79^. In fact, the high mean net photosynthesis of 0.51 g C m^−2^ h^−1^ measured in Area A in summer, when precipitation is non-existent and temperatures often exceed the theoretical optimal 35 °C for mangrove carbon assimilation^80^, lends some further support to this idea.

Mangrove forest conservation can benefit from carbon assimilation maps produced by remote sensing, which can in turn help to inform decision-making processes^81^ and improve communication between stakeholders and scientists^82^. By providing high spatiotemporal insights into vegetation dynamics, novel remote sensing platforms with high revisit times and spatial resolution, such as CubeSats^83^, can further advance mangrove conservation and restoration efforts. Future studies will need to determine if the results obtained herein are consistent when using sensors with coarser spatial resolutions (relative to high-resolution UAV data). Nevertheless, remote sensing data should be leveraged with field measurements and UAVs can be used as a stepping stone to better understand the error that might be propagated in the process^84^. Moreover, subsequent research seeking to estimate the carbon assimilation of mangroves over larger, regional scales will benefit from natural synergies between data collected from UAV and satellite platforms. That is, the UAV data can be used for training or scaling-up point-scale measurements and serve as an intermediate step for using satellite image data to cover larger spatial extents^83,85^. Studies evaluating uncertainties when scaling between UAV- and satellite-derived datasets will be required to understand error propagation when fusing these types of data together^84^, but ultimately leading to improved estimates of carbon assimilation.

Mangroves in the Red Sea are mainly dominated by *A. marina*, but stands of *R. mucronata* are also present^86,87^ with different soil carbon sequestration rates^88^. As such, there is a need to evaluate the rates of carbon assimilation of these two species both here and in other regions of the world (e.g.,^36^). Additional work should evaluate if the carbon assimilated by stands in which these two species appear together can be assessed by using a similar regression approach to the one presented herein.

Although regression models have been widely used to estimate the LAI of mangroves using remote sensing data (e.g.,^36,89^), they are only as reliable as their training data. Therefore, choosing datasets that capture the range of ecosystem variability is important to ensure model representativeness: especially if applied beyond the studied domains. The mangrove forests evaluated in this work were located within a few kilometers of each other and grow under similar environmental conditions, increasing the likelihood of achieving similar models for all three sites. Additionally, the Red Sea’s minimal seasonality enables mangroves to maintain nearly constant physiology, which facilitates the derivation of consistent results from different stands throughout the year. As such, additional research should evaluate whether the model developed herein is applicable to mangrove forests in other parts of the Red Sea that might be exposed to different environmental conditions. It is at this regional level of detail that satellite data can provide the information required for national agencies interested in regional and even country-wide conservation, planning programs, climate change mitigation strategies, and offsetting other carbon-producing activities^90^. Furthermore, regional estimates of carbon sequestration might help to improve global carbon budget estimates by accounting for regional variability. Future work should explore other modeling approaches (e.g., machine and deep learning methods) to assess if the estimation of LAI can be further improved, as investigated in previous works (e.g.,^91^).

The instrument used to estimate LAI (i.e., LiCOR LAI-2200C) measures diffuse radiation transmissions and does not distinguish between active leaf tissue and other plant parts, such as stems or branches^92^, which can influence LAI values when the foliage is not uniform^93,94^. Additional analysis should evaluate if different measurements of LAI affect the relationships observed in this work between LAI and carbon assimilation. In addition, future research should assess whether the proposed methodology can effectively evaluate spatial and temporal changes in carbon assimilation rates in mangroves, which may vary depending on the season. Further, ongoing investigation should compare our results with those obtained from eddy covariance methods, which have been applied in mangroves worldwide, but are scarce in arid environments (e.g.,^27,95^). As the gold standard in large-scale carbon flux monitoring^24^, eddy-covariance methods might provide more accurate measurements of canopy-scale ecosystem function than the point-scale gas exchange chamber used herein.

## Conclusions

The feasibility of quantifying carbon assimilation from UAV-based multispectral data trained by field measurements of LAI and net photosynthesis was explored. As a first step, multispectral UAV imagery was leveraged to estimate the leaf area index (LAI) across three different mangrove sites using a multiple linear regression approach. Subsequent analysis demonstrated the potential of using these UAV-derived LAI data together with field observations of net photosynthesis to produce spatial maps of carbon assimilation. Our carbon assimilation estimates demonstrate that Red Sea mangroves, mainly dominated by *A. marina,* have a similar capacity to assimilate carbon compared to *A. marina* stands in other geographical regions (e.g.,^36,67^). Expanding our understanding of the physiology of mangroves in the Red Sea increases both our capacity to preserve and protect mangroves, and also provides the scientific guidance required to advance them as a potential nature-based solution. As demonstrated herein, remote sensing has a fundamental role to play in upscaling ground-based measurements: particularly those that can be used to assess carbon assimilation via mangroves. With this knowledge, both local and regional scale investigations of mangrove health and condition, and an exploration of their role as a carbon offsetting mechanism can be more accurately quantified.

### Supplementary Information

Supplementary Information.

## Data Availability

The data that support the finding of this study are available from the authors upon reasonable request and with permission of KAUST.
