# Supplementary material for: Quantifying mangrove carbon assimilation rates using UAV imagery
Source: Sci Rep. 2024 Feb 26;14:4648. doi: 10.1038/s41598-024-55090-w (PMC10897312; doi:10.1038/s41598-024-55090-w)
Supplement: Supplementary file 1 — Supplementary Information. [file 41598_2024_55090_MOESM1_ESM.docx]

**Supplementary material**

$$\boldsymbol{P}_{\boldsymbol{N}}\boldsymbol{=A*}\frac{\boldsymbol{\mu mol}\boldsymbol{CO}^{\boldsymbol{2}}}{\boldsymbol{m}^{\boldsymbol{-2}}\boldsymbol{s}^{\boldsymbol{-2}}}\boldsymbol{*}\frac{\boldsymbol{1 mol}\boldsymbol{CO}^{\boldsymbol{2}}}{\boldsymbol{1*}\boldsymbol{10}^{\boldsymbol{6}}\boldsymbol{\mu mol}\boldsymbol{CO}^{\boldsymbol{2}}}\boldsymbol{*}\frac{\boldsymbol{1 mol C}}{\boldsymbol{1 mol}\boldsymbol{CO}^{\boldsymbol{2}}}\boldsymbol{*}\frac{\boldsymbol{12 g C}}{\boldsymbol{1 mol C}}\boldsymbol{*}\frac{\boldsymbol{1 kg C}}{\boldsymbol{1*}\boldsymbol{10}^{\boldsymbol{3}}\boldsymbol{g C}}\boldsymbol{*}\frac{\boldsymbol{LAI}\boldsymbol{m}^{\boldsymbol{2}}}{\boldsymbol{m}^{\boldsymbol{2}}}$$

$$\boldsymbol{*}\frac{\boldsymbol{1}\boldsymbol{m}^{\boldsymbol{2}}}{\boldsymbol{10}\boldsymbol{*10}^{\boldsymbol{3}}\boldsymbol{cm}^{\boldsymbol{2}}}\boldsymbol{*}\frac{\boldsymbol{11.6 h}}{\boldsymbol{1 d}}\boldsymbol{*}\frac{\boldsymbol{3600 s}}{\boldsymbol{1 h}}\boldsymbol{*}\frac{\boldsymbol{365 d}}{\boldsymbol{1 yr}}$$

**Equation S1**. Conversion applied to the carbon assimilation from μmol CO_2_ m⁻² s⁻¹ to kg C pixel⁻¹ yr⁻¹. P_N_: net canopy photosynthesis. A: average net photosynthesis. LAI: leaf area index.


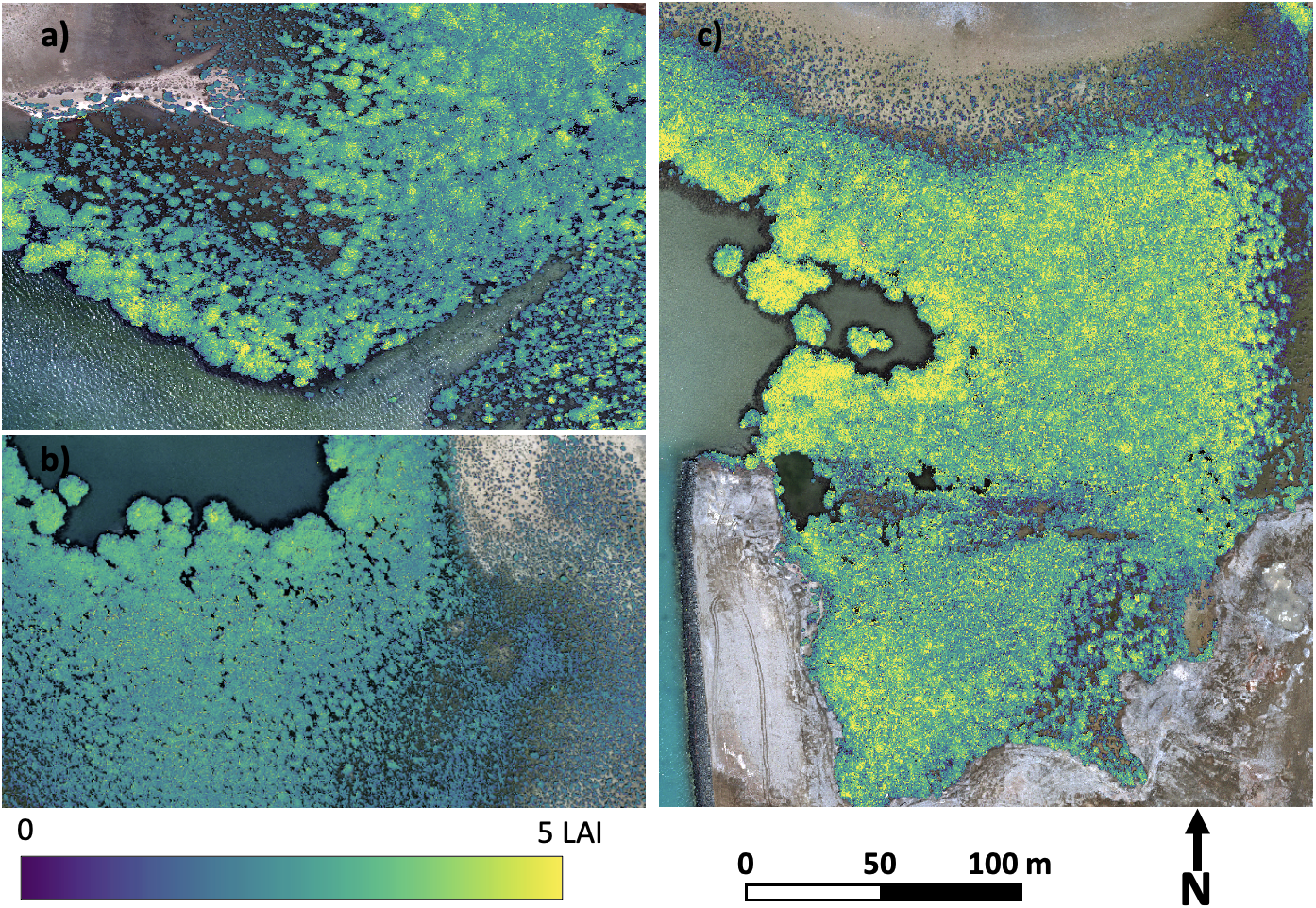


**Figure S1**. Maps of estimated leaf area index (LAI) derived from multispectral unmanned aerial vehicle imagery for a subset of: a) Area A; b) Area B; c) Area C.
